# Oleo-Brassidate of Mercury Inunctions

**Published:** 1909-08-21

**Authors:** 


### OLEO-BRASSIDATE OF MERCURY INUNCTIONS.

Colza oil contains erucic acid amongst its other constituents, and erucic acid can be isomerised into brassidic acid. The latter can then be treated with a mixture of mercuric oxide and oleic acid, with the result that a substance known as mercuric oleo-brassidate is produced. This is readily soluble in soap solution, and it can be spread and applied to the skin without giving so greasy a feeling as ordinary ointments do; moreover, it is said to be very readily absorbed, so that it is of use for purposes of mercurial inunction in syphilis and other affections. Doses up to as much as 270 grains in a day have been applied daily or every other day, and stomatitis has occurred in one case only even when as many as thirty applications have been made. The method of inunction is simply that of steady persistent rubbing without any force or severity. The preparation is equally serviceable as a local antiseptic application in conditions such as pediculosis vestimentorum, scabies, tinea cruris, and other similar affections. One great advantage in the mercuric oleo-brassidate is its ready absorbability, so that linen and underclothing escape that oily saturation that is almost unavoidable when ordinary ointments are used.

